# Spatial Learning in Japanese Eels Using Extra- and Intra-Maze Cues

**DOI:** 10.3389/fpsyg.2020.01350

**Published:** 2020-07-16

**Authors:** Shigeru Watanabe

**Affiliations:** Department of Psychology, Keio University, Tokyo, Japan

**Keywords:** spatial cognition, visual discrimination, intra-maze cue, extra-maze cue, attention

## Abstract

Japanese eels (*Anguilla japonica*) were trained on a spatial-learning paradigm in a pool placed in an experimental room where several extra-maze cues were present. Four tubes were placed in the pool, of which one was open and could be entered by the eels. The open tube was placed at a fixed position in the pool and contained a triangular block that served as an intra-maze cue. The eels learned to identify the open tube, and their performance was maintained when the pool was rotated. However, they were unable to maintain their performance in a dark room, which suggests that spatial learning is based on visual cues. To determine the influence of the extra- and intra-maze cues, the tube with the triangle was moved to a new position and another open tube was kept in its place. The eels chose either the tube at the original position or the tube with the triangle at its new position, suggesting that spatial discrimination may be based on either extra- or intra-maze cues. We thus conclude that the eels employed an adjunctive strategy of multiple cues. In the next experiment, the eels were trained to visually discriminate the position of the stimulus (triangle), which changed in every trial. After the training, the eels were submitted to a test in which, in addition to the triangular pattern, a rectangular pattern was introduced. The eels discriminated between the tubes with the triangular and rectangular patterns, suggesting that they had the ability to discriminate visual patterns.

## Introduction

Animals living in the hydrosphere are important subjects for studies on comparative psychology because more than 26000 species of the 45000 vertebrates on earth live underwater. To find a general rule of learning or to find divergence in learning abilities, studying the behavior of animals in the hydrosphere is imperative. MacPhail (1985) hypothesized that there is no difference between the intellects of non-human vertebrates. Recent studies on fish demonstrated a variety of higher cognitive abilities comparable to those of mammals, including self-recognition in mirrors (Kohda et al., 2019), sense of numbers (Agrillo et al., 2012), human face recognition (Newport et al., 2018), transitive inference (Grosenick et al., 2007), or episode-like memory (Hamilton et al., 2016). Some fish also displayed complex architecture comparable to that of bower birds (Matsuura, 2015).

Some fish show remarkable orientation and navigating abilities for migration (Dodson, 1988; Braithwaite and Burt de Perera, 2006) and others live in complex environments that require them to have considerable spatial memory (Brown, 2015). The jumping goby (*Bathygobius soporator*), for instance, swims over tidal areas during high tides and apparently learns the topography of the area. Aaronson (1971) constructed an artificial tide pool and confirmed that this species learned spatial configuration during high tides and used it to jump to safe tide pools during low tides. This study thus demonstrated that fish are capable of spatial learning.

The radial arm maze was designed by Olton and Samuelson (1976) to measure spatial learning and memory in rats. Roitblat et al. (1982) trained Siamese fighting fish (*Betta splendens*) in an eight-armed maze, and they found that the fish made an average of 6.63 successful choices in eight attempts; they also inserted a 5-min delay between the fourth and fifth choice, which reduced the overall success rate. Hughes and Blight (1999) performed an experiment using two intertidal species, *Spinachia spinachia* and *Crenilabrus melops*, which belong to different families but live in similar habitats. Without any extra-maze spatial cues, both species showed a fixed pattern while visiting the arms; however, they followed a spatial memory-related strategy when visual intra-maze cues (i.e., colored tiles on the floor) were present. Rotating the maze did not impair their foraging behavior, but rearranging the tiles did; therefore, Hughes and Blight (1999) suggested that the fish used tile configuration as a cue for spatial learning. These species also associated visual cues with food location in the radial maze (Hughes and Blight, 2000).

Another common apparatus used in spatial memory experiments on rodents is the Morris water maze (Morris, 1981). An apparatus for spatial learning in goldfish, similar to a dry version of the Morris maze, was developed by Saito and Watanabe (2005). This maze had 16 small holes, one of which was baited. The study showed that, after the goldfish learned the position of the baited hole, rotating the maze did not affect the fish’s performance, whereas covering the maze with a curtain disrupted their performance. Moreover, sectioning the fish’s olfactory nerves did not impair performance, but eye enucleation did. These results led the authors to conclude that goldfish used visual extra-maze cues for spatial learning. In another experiment (Saito and Watanabe, 2004), a landmark was placed in the maze. The study showed that, even when the landmark and food positions were changed every day, as long as the spatial relation of the two with reference to each other was fixed, the fish were able to learn this task using the visual intra-maze cue. Durán et al. (2008) also reported intra-maze cue-learning in goldfish. These results demonstrated that fish have spatial-learning abilities comparable to those of rodents.

Eels have an outstanding migratory ability. Tsukamoto et al. (2011) found hatched eggs and Japanese eel larvae in the west Mariana Ridge, which indicates that larvae are able to migrate from this location to Japan and that adult eels can swim thousands of kilometers back to the west Mariana Ridge. Studies have examined the sensory systems of eels, such as olfaction (Westin, 1990; Tesch et al., 1991; Barbin et al., 1998; Huertas et al., 2010; Churcher et al., 2015; Schmucker et al., 2016), magnetic sense (Nishi et al., 2005; Cresci et al., 2017; Naisbett-Jones et al., 2017), and vision (Omura et al., 1997; Byzov et al., 1998). The retina of Japanese eel larvae has cones (Omura et al., 1997), and Byzov et al. (1998) reported yellow-sensitive and green-sensitive cones in European eels (*Anguilla anguilla*), indicating that these animals have color vision. However, except for the study by Watanabe and Shinozuka (2020), the spatial-learning ability of eels has not yet been examined in a laboratory.

Setting up a suitable apparatus to examine spatial learning in a new species is crucial. A possible apparatus that could be used for eels is the one used for octopi. Boal et al. (2000) released an octopus in a pool with five closed and one open burrow; the octopus learned the position of the open one. This apparatus is functionally similar to the Morris maze. As eels prefer to hide in small holes such as tubes, we examined spatial learning in eels using this habit as reinforcement (Watanabe and Shinozuka, 2020). Four tubes were placed in a round pool within an experimental chamber with several extra-maze cues. One tube, placed at a fixed position, was open, and the other tubes were closed. When the eel reached the open tube, it was allowed to stay in there for 10 min; thus, they learned the position of the open tube. The eels were unable to maintain their discriminative behavior when the test was performed in a dark room. These results demonstrated that spatial learning in eels was based on extra-maze visual cues. Natural settings commonly have both extra- and intra-maze cues. Therefore, in the present study, eels were trained in a pool with both extra- and intra-maze cues, and their spatial discrimination based on these cues was examined.

## Experiment I: Maze With Extra-and Intra-Maze Cues

### Materials and Methods

#### Study Subjects

Nine Japanese eels (*Anguilla japonica*), obtained from Omori-Tansui Co., Ltd. (Miyazaki, Japan), were used in this experiment. The length of the eels was 22–35 cm. The eels were housed individually in aquaria (39.8 × 25.4 × 28 cm) that were filled with dechlorinated tap water and fitted with an air pump. Sand was placed on the floor of each aquarium, and a gray vinyl chloride tube (inner diameter, 1.5 cm; length, 24 cm) was added to each aquarium. The experiments were initiated 2 weeks after the eels were brought to the laboratory. A 13L:11D artificial illumination cycle was used, but the racks holding the aquariums were covered with a gray vinyl curtain. Earthworms were provided once a week, but most of the eels did not eat them.

#### Apparatus

**FIGURE 1:**
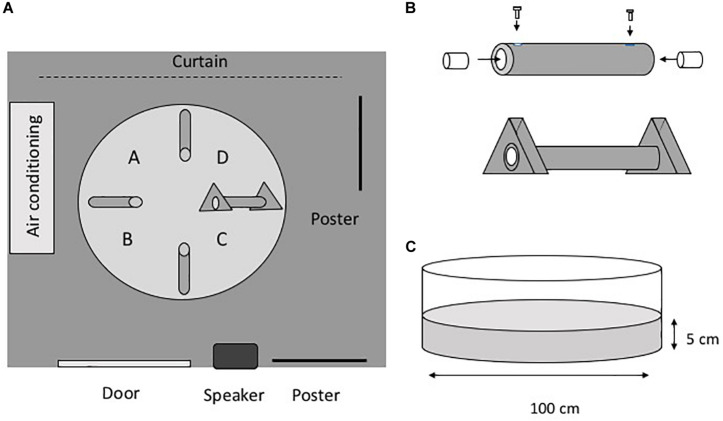
Apparatus. **(A)** Plain view of the experimental room. The room has several extra-maze cues, such as posters and furniture. A–D indicate the place of release of the subjects. **(B)** Tube. To prepare the closed tubes, a small acyl cylinder was fixed at both ends of the tube. The distance from the entrance of the tube to the cylinder is 1.0 cm. The cylinders are movable in the open tubes. A triangular block was fixed at both ends of the open tube. **(C)** Pool.

Figure 1 shows the experimental setup and apparatus. The experimental maze consisted of a white polypropylene circular pool (diameter, 100 cm; depth, 38 cm) filled with dechlorinated tap water (Figure 1). The water level was 5 cm from the bottom of the pool. The water temperature was maintained at 25°C, and the water was changed every fifth day. The experimental room was illuminated with fluorescent lamps, and there were several extra-maze cues in the room (Figure 1A). The pool contained four gray vinyl chloride tubes (inner diameter, 1.6 cm; length, 24 cm), and each tube had four lead weights (20 mm^3^) attached to it to affix it to the floor. A transparent acyl cylinder (diameter, 15 mm; length, 30 mm) was inserted into both ends of the tubes. For the three closed tubes, acyl screws fixed the acyl cylinders so that the eels could not enter the tube. For the open tube, the acyl cylinders were not fixed with screws so that the eels would be able to enter the tube. A gray vinyl chloride equilateral triangle (base, 80 mm; thickness, 30 mm) was fixed at both ends of the open tube. Eel behavior was monitored using a CCD camera (G100, NEC Avio, Tokyo, Japan) connected to a computer. In one of the tests, which was carried out in a dark room, a night scope (Super Night Compact 1000 NDX; Kenko Tokina Co., Ltd., Tokyo, Japan) was also used to observe the eels.

#### Habituation to the Apparatus

Each eel was individually habituated to the apparatus. An eel was first transferred from its aquarium to a bucket using a nylon net. Then, it was gently released from the bucket into the pool. During the habituation phase, all the tubes were open and no triangles were attached to any tubes. The eel was allowed to move around the pool for 10 min during which time it typically selected one tube and stayed inside it. At the end of the 10 min, the eel was returned to the aquarium. All the tubes were cleaned with a brush before the next eel was released into the apparatus. This procedure was repeated for 2 days.

#### Spatial Discrimination Training

During the spatial discrimination training phase, the treatment of the eels was identical to that during the habituation phase, except that only one tube with a triangle at a fixed position was open. An eel could visit the tubes until it reached the open tube. When the eel entered the open tube, it was allowed to stay there for 10 min. The first choice was recorded as its response in that trial. If the eel did not enter the tube, the eel was retrieved after 10 min. Because catching and releasing causes stress in the eels, all eels underwent only one training trial per day, and the position at which they were released was randomly assigned (Figure 1). The eels had to undergo at least 10 training trials, and the criterion used to determine if the eel was trained was three correct responses within four successive trials (*P* = 0.004, binominal test). Once trained, the following tests were performed.

#### Rotation Test (Three Trials)

The eels might use unknown visual cues inside the pool to learn the position of the correct tube. To prevent the eels from using such cues, the pool was rotated 90°, 180°, or 270° at each trial. Each eel underwent only one trial at each of the three rotation positions. The open tube remained at its original position relative to the room. Again, each eel underwent one trial test per day.

#### Dark Room Test (Four Trials)

To eliminate visual cues, a test similar to that of the spatial discrimination training was carried out in a dark room. The leaked illumination was measured from nine positions inside the pool. Mean illuminance was 0.10 Lx in the dark room, whereas that in the illuminated room was 368 Lx. The test was repeated four times for each eel, with one trial per day.

#### Cue Separation Test (Three Trials)

In this test, the local cue (triangle) and global cue (the position of the open tube) were separated. The tube with the triangle was moved to one of three new positions. At its original position, another tube was placed. The tube with the triangular cue and the newly placed tube were both open so that eels could enter either of them. The test procedure was similar to that of the spatial discrimination training, except that there were two open tubes. The test was repeated three times; at each trial, the position of the tube with the triangular cue was changed.

#### Bidimensional (2D) Triangle Test (Four Trials)

In this test, the triangular blocks had tridimensional (3D) stimuli; i.e., not only visual cues but also tactile cues could be used. In this test, a triangular pattern, similar in shape, color, and size to that of the original triangular block, was pasted on an 80 × 80 mm transparent acyl board, and the board was fixed at both ends of the open tube. Transparent acyl boards without patterns were fixed on the closed tubes. The test consisted of four trials.

#### Statistical Procedures

A single sample *t*-test was used for the analysis of cumulative correct responses in each test. One-factor ANOVA was used for comparison among tests, and Shaffer’s modified sequentially rejective Bonferroni procedure was used for *post hoc* multiple comparisons.

### Results

**FIGURE 2:**
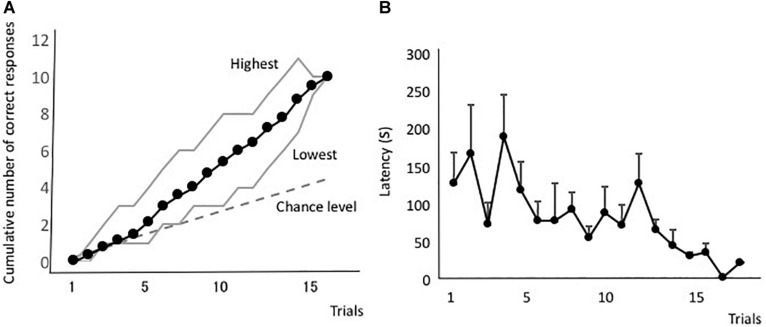
Forward-learning curves. **(A)** The vertical axis indicates the cumulative number of correct responses. “Highest” and “Lowest” indicate the highest and lowest scores at each trial. The black line indicates the average value, the broken line indicates the expected cumulative number, and gray lines indicate the highest and lowest number at each trial. **(B)** Average time taken from release to the point of entry into the open tube. Latency of subjects that did not enter the tube is assigned as 600 s.

Figure 2A shows the averaged forward-learning curve. The vertical axis indicates the cumulative number of correct responses. The eels that achieved the discrimination criterion (i.e., three correct responses in four consecutive trials) were not considered for averaging the calculations of cumulative correct trials. All eels reached the discrimination criterion. The fastest an eel took to reach the criterion was after 10 trials, whereas the slowest was after 18 trials (average: 14.3 trials). Figure 2B shows the averaged latency. The latency to reach the open tube decreased throughout the trials. The means of the first and last four trials were 140 s (sd = 83.4) and 66.1 s (sd = 35.8), respectively, and they were significantly different [two-tailed paired *t*-test, *t*(8) = 2.56, *P* = 0.03]. Thus, the eels learned how to detect the correct tube quickly.

**FIGURE 3:**
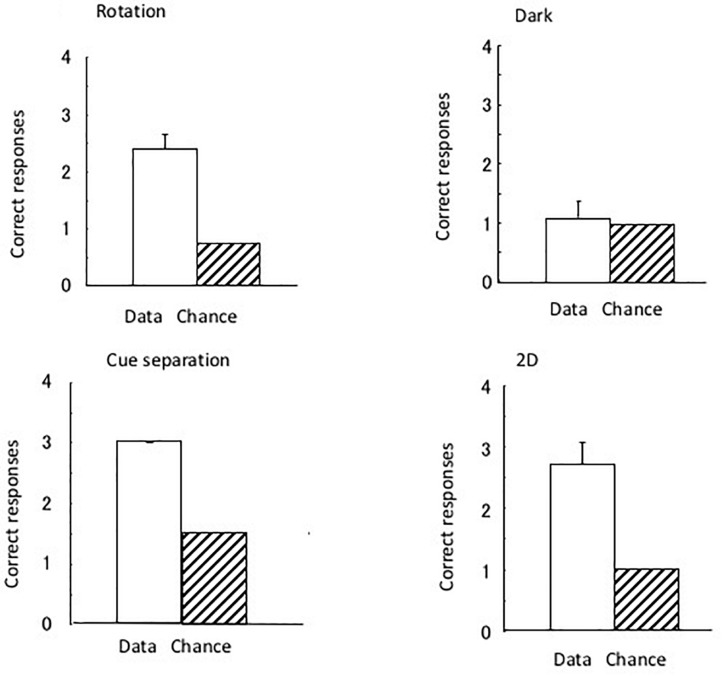
The results of the four tests conducted under Experiment I. Open bars and hashed bars indicate the data and chance level in each test. Small bars indicate the standard error. None of the subjects showed an error in the separation test.

Figure 3 shows the results of the four tests that were performed. As four trials were performed for the dark test and three for the rotation and 2D tests, the chance of the eels having a successful performance was 1.25 and 1.0, respectively. As there were two correct (i.e., open) tubes (i.e., one with the correct pattern and one at the correct position) in the cue separation test, the chance of having a successful performance in three trials was 1.5. The subjects clearly maintained their discriminative behavior in all tests, except in the dark room test. In the total of 36 dark test trials, a subject did not reach the open tube within 10 min in only 1 trial. The subjects demonstrated perfect performance in the cue separation test. Therefore, the eels detected the open tubes when the local pattern cue and global position cue were separated. Single sample *t*-tests revealed a significant difference in the chance level of the rotation [*t*(8) = 7.0, *P* < 0.001] and 2D tests [*t*(8) = 5.57, *P* < 0.001] but no significant difference in the dark test [*t*(8) = 0.43, *P* = 0.68]. They did not use possible visual cues, such as small scratches on the wall of the pool, to identify the open tube, and they used visual rather than tactile cues to identify the triangular block. The results of the dark test demonstrated that the eels were unable to maintain their discriminative behavior without visual information.

**FIGURE 4:**
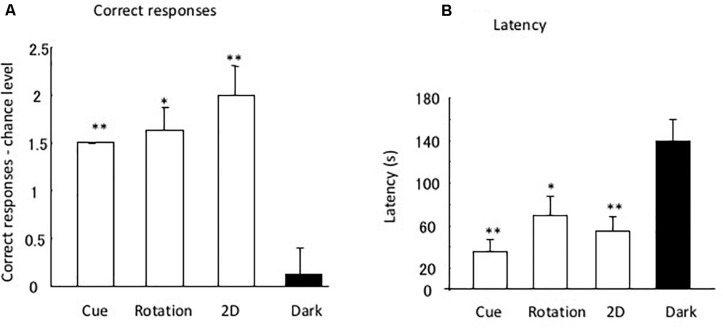
Difference between data and chance level. **(A,B)** Indicate the correct response and latency, respectively. Small bars indicate the standard error. *P < 0.05, **P < 0.01.

Figure 4A shows the differences in the chance level for each test. One-factor ANOVA indicated the significant effects of the tests [*F*(3,31) = 12.90, *P* = 0.0001]. *Post hoc* multiple comparison showed a significant difference between the dark test and the rotation [*t*(7) = 4.66, *P* = 0.007], 2D test [*t*(7) = 3.64, *P* = 0.025], and cue separation tests [*t*(7) = 15.0, *P* < 0.0001]. No significant difference was found for the other combinations.

Figure 4B shows the mean latency to reach the open tube in the four tests. Analysis of latency demonstrated that the eels required a longer time to reach the open tube in the dark than under light conditions. One-factor ANOVA indicated the significant effects of the tests [*F*(3,31) = 10.81, *P* = 0.0002]. The *post hoc* multiple comparison showed a significant difference between the dark and rotation tests [*t*(7) = 4.03, *P* = 0.01], between the dark and cue separation tests [*t*(8) = 3.64, *P* = 0.025], and between the dark and 2D tests [*t*(7) = 5.00, *P* = 0.009]. No significant difference was found for the other combinations.

**FIGURE 5:**
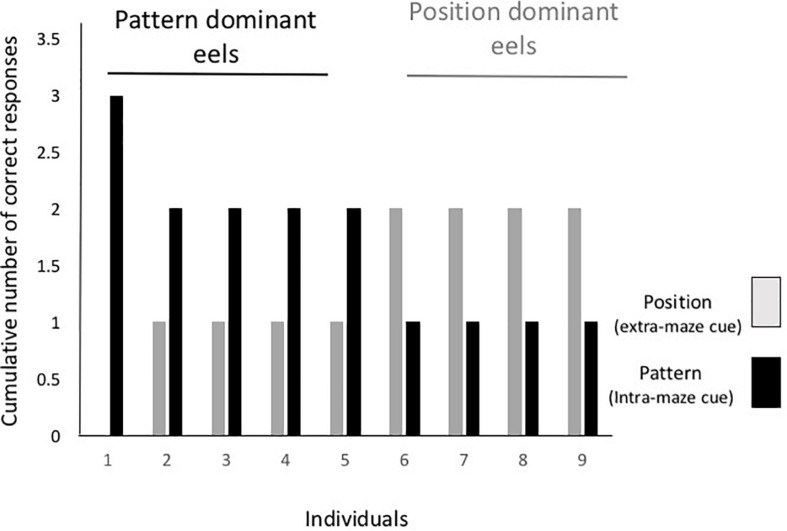
Individual results from the cue separation test. Gray and black bars indicate the number of trials required until either the tube at the correct position or the tube with the correct pattern was chosen.

Figure 5 shows the individual data from the cue separation test. One individual selected the tube with a triangle in all the test trials regardless of the tube’s position, and four other eels chose the tube with the triangular cue in only two trials. The other four eels chose the tube at the correct position in two trials and that with the triangle in one trial. Thus, there was no consistent dominant cue among individuals, and cue selection varied with individual and trial.

In the first trial of the test, five eels selected the spatial cue and four selected the pattern cue. Five eels chose the same cue in the first and second trials, and three eels changed the cue in the first and second trials. Thus, no tendency regarding discriminative behavior from the first trial to the last trial was detected.

## Experiment II: Visual Discrimination

Experiment I demonstrated that eels used not only the global positional cue but also the local visual cue for spatial discrimination. In Experiment II, the eels were trained to visually discriminate the position of the 2D stimulus (triangle), which changed in every trial. Thus, they had to learn the correct tube by the intra-maze cue alone. After the discriminative training, the eels were subjected to a test in which a triangular and a rectangular pattern were introduced. If the eels identify the correct tube as a tube with a darker board, they should choose either the tube with the triangular pattern or that with the rectangular pattern. Contrastingly, if the stimulus control by the triangle is visual pattern discrimination, the eels should choose the tube with the triangular pattern.

### Materials and Methods

#### Subjects

Five eels from Experiment I were used for this experiment.

#### Apparatus

This experiment used the same apparatus as in Experiment I. The open tube had triangular boards at both ends. The other three tubes had transparent boards.

#### Procedure

The subjects were trained on visual discrimination using a tube with a triangular board and closed tubes with transparent acyl boards without a triangle. The tube with the triangular cue was placed at a different position in each trial. The following procedure was identical to that performed in Experiment I. The subjects were trained for at least 10 trials. The subjects that made three correct choices in four consecutive trials were used for the test, whereas those that failed to reach the criterion in 10 trials received additional training until the criterion was reached.

#### Triangle Versus Rectangle Test

A 2D triangle was fixed to the open tube, whereas one of the three closed tubes had a 2D rectangular pattern. For the latter, a gray rectangular pattern (80 × 80 mm) was pasted on an acyl board, and this board was fixed at each end of the closed tube. The other two closed tubes had transparent boards. The test consisted of four trials, and the positions of the open tube with the triangular cue and the closed tube with the rectangular cue were quasi-randomly assigned. There was no repetition of the same arrangement for both tubes.

### Results

**FIGURE 6:**
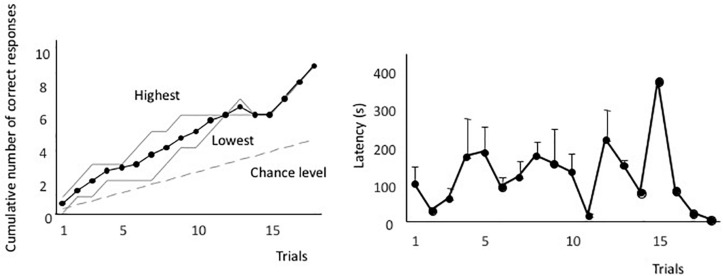
Forward-learning curve for Experiment II **(A)**, and forward mean of the latency **(B)**.

Figure 6A shows the forward-learning curves resulting from this test. Three eels reached the criterion in 10 trials, one in 13 trials, and one in 18 trials. Thus, the eels were able to learn visual discrimination. Latency during the discriminative training is shown in Figure 6B. There was no clear improvement in latency. The mean of the first and last four trials was 88.8 s (sd = 52.7) and 110.3 s (sd = 41.9), respectively. There was no statistically significant difference between them [two-tailed paired *t*-test, *t*(4) = 0.10, *P* = 0.93].

**FIGURE 7:**
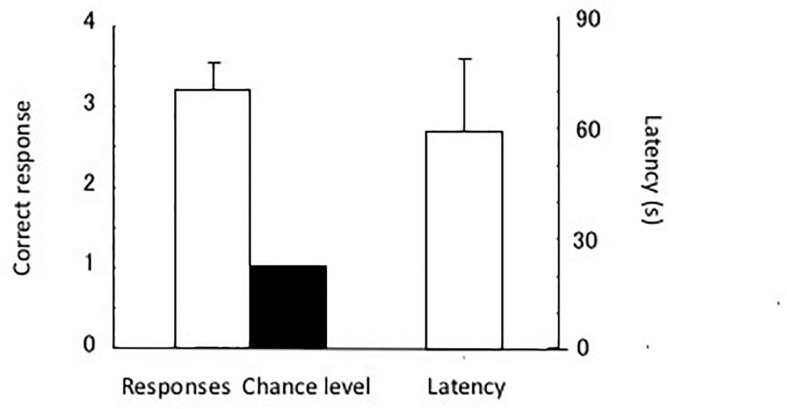
Results of the test. Correct response **(left)** and latency **(right)**. Small bars indicate the standard error.

The test results are shown in Figure 7. The eels chose the tube with the triangular cue. The single sample *t*-test showed a significant difference in chance level [*t*(4) = 5.88, *P* < 0.005]. The eels chose a total of four incorrect tubes in the 20 test trials, and two of the incorrect choices were regarding the tube with the rectangular cue. Thus, they were slightly distracted by the rectangular pattern. In other words, the shape of the stimulus (triangle) controlled the discriminative behavior of the eels.

## Discussion

The results of the present study show that: (1) the eels used visual cues for spatial learning; (2) they used extra- and intra-maze cues; and (3) they were able to learn visual discrimination without spatial cues.

### Comparison With Other Studies

The eels’ performance in the experiments show the effectiveness of using their behavior of hiding in a shelter tube as reinforcement, and these results confirm previous findings (Watanabe and Shinozuka, 2020). A previous experiment showed that the eels’ spatial-learning ability was based only on extra-maze cues (16.4 trails, on average). We herein show that the eels’ spatial-learning ability was based on both extra- and intra-maze cues (14.3 trials, on average). Although the eels seem to have had more success in the present study than in the previous one, no statistically significant difference was found between these results [two-tailed *t*-test, *t*(19) = 1.13, *P* = 0.27]. Therefore, adding intra-maze cues did not significantly improve the acquisition of spatial learning.

Because of the different methodologies used, a comparison of the present results from experiments on eels with the results from other studies on other fish species is rather difficult. Goldfish, for instance, demonstrated spatial-learning ability in a Morris-type maze with extra- or intra-maze cues after 35.2 trials on average and 22.4 trails (four trials per day), respectively (Saito and Watanabe, 2004). Moreover, the goldfish completed the Morris-like maze task with both extra- and intra-maze cues faster than that without intra-maze cues (Saito and Watanabe, 2005). Taniuchi et al. (2013) also reported the slight positive effect of intra-maze cues on the performance of goldfish in a radial maze task. These results suggest the additive effect of intra- and extra-maze cues in goldfish.

Octopi trained to find an open burrow showed a clear decrease in the distance traveled to find the open burrow within six trials (Boal et al., 2000). Although the data on the success rate is not available and the number of choices is different from that in the present experiment, the performance of the octopi seems comparable to that of fish. Zebra finches trained to choose one of four feeders in a flying cage acquired spatial learning in nine to twelve trials (Watanabe and Bischof, 2002, 2004). Although differences in the reinforcement used and the number of trials per day must be considered, this suggests that spatial learning in eels is comparable to that of zebra finches. C57/BL mice learned the standard Morris water maze in 15–20 trials (Yoshida et al., 2001). They also learned a dry-type Morris maze with auditory extra-maze cues (Watanabe and Yoshida, 2007) and one with airflow cues (Bouchekioua et al., 2014) in 15–20 trials. Therefore, successful learning of similar spatial tasks in eels, goldfish, octopus, zebra finch, and rodents supports the null hypothesis proposed by MacPhail (1985).

### Visual Information and Spatial Learning

The results of the rotation and dark tests suggest that eels learned spatial discrimination based on visual cues, which confirms our previous findings (Watanabe and Shinozuka, 2020). The eels in the present experiment showed a slow response in the dark test. Although the swimming speed might be reduced in the dark, visiting incorrect tubes was the main factor prolonging the latency. In 35 of 36 cases, the eels finally reached the correct tube after visits to the wrong tubes. Thus, the chance level performance in the dark test can be attributed to a lack of discriminative behavior rather than motor deficits. Goldfish also maintained spatial discrimination after the rotation of the experimental pool, but spatial learning was lost after enucleation of the eyes (Saito and Watanabe, 2004).

The 2D test performed in Experiment I suggests that the visual cue was more influential than the tactile cue. As vision is a sense that detects distance, visual cues may be more effective in the detection of hiding places from a distance. Since the work performed by Sutherland (1969), many other studies on fish visual discrimination have been performed and have found that some fish species are capable of fine visual discrimination (e.g., human face discrimination performed by Archer fish, *Toxotes chatareus*; Newport et al., 2018). Unfortunately, no other data on eel visual discrimination is available. However, the results of Experiment II demonstrated that eels can learn to visually discriminate shapes.

### Cue Selection in Spatial Learning

In this study, the experiments provided the eels with redundant extra- and intra-maze cues to detect the correct tube. Selective attention occurs when an animal uses one particular cue and ignores the other cues (Reynolds, 1961), whereas divided attention occurs when an animal processes two or more elements of compound stimuli (see Zentall, 2005 for review). The results of the present study suggest that most of the eels used both extra- and intra-maze cues rather than selective attention. The dominancy of a cue depends on its salience or on the discriminability of the cues; therefore, it is premature to presume the total absence of selective attention in eels based on the present experiment alone. Although the intra-maze cues did significantly affect the eels’ discriminative behavior, the eels’ perfect performance in the separation test suggests that they learned the tasks as an adjunctive of the extra- and intra-maze cues rather than as a conjunctive of them. They learned both cues during the training, and either cue type may have provided them with enough information to detect the correct tube. When the subject finds the intra-maze cue first, it is expected to choose the correct pattern tube, and when it identifies enough extra-maze cues first, it is expected to choose the correct position tube. The perfect performance and the maintenance of the latency in the cue separation test also support this adjunctive strategy.

### Limitation and Future Studies

The present experiment used hiding behavior as a reinforcement of training. Thus, the spatial learning here might be a result of a particular form of training from a particular reinforcement and not a general ability of eels.

Eels are a novel subject in comparative psychology. Basic research, such as psychophysics and general-learning ability, should be done to understand their behavior. Eels are easy to maintain in a laboratory, and they may be used as possible experimental models for studies on spatial cognition. As their navigational abilities in natural settings are outstanding, behavioral studies on eels performed in a laboratory can help bridge our understanding of spatial cognition not only in the laboratory but also in field studies. Furthermore, eels are also important in fishery science. Obtaining an understanding of eel behavior will contribute to the preservation of this animal.

## Data Availability Statement

All datasets generated for this study are included in the article/Supplementary Material.

## Ethics Statement

Ethical review and approval was not required for the animal study because Fish are not target of our animal experiment committee. There was no deprivation nor physical invasion at all.

## Author Contributions

SW designed the study and conducted the experiments, collected the data, analyzed the data, and wrote the manuscript.

## Conflict of Interest

The author declares that the research was conducted in the absence of any commercial or financial relationships that could be construed as a potential conflict of interest.
